# Short Implicit Voice Training Affects Listening Effort During a Voice Cue Sensitivity Task With Vocoder-Degraded Speech

**DOI:** 10.1097/AUD.0000000000001335

**Published:** 2023-01-25

**Authors:** Ada Biçer, Thomas Koelewijn, Deniz Başkent

**Affiliations:** 1 Department of Otorhinolaryngology/Head and Neck Surgery, University Medical Center Groningen, University of Groningen, Groningen, The Netherlands; 2 Research School of Behavioural and Cognitive Neurosciences, Graduate School of Medical Sciences, University of Groningen, Groningen, The Netherlands.

**Keywords:** Listening effort, Vocal-tract length, Voice cue perception, Voice familiarity, Voice pitch, Voice training

## Abstract

Understanding speech in real life can be challenging and effortful, such as in multiple-talker listening conditions. Fundamental frequency (*f*_*o*_) and vocal-tract length (*vtl*) voice cues can help listeners segregate between talkers, enhancing speech perception in adverse listening conditions. Previous research showed lower sensitivity to *f*_*o*_ and *vtl* voice cues when speech signal was degraded, such as in cochlear implant hearing and vocoder-listening compared to normal hearing, likely contributing to difficulties in understanding speech in adverse listening. Nevertheless, when multiple talkers are present, familiarity with a talker’s voice, via training or exposure, could provide a speech intelligibility benefit. In this study, the objective was to assess how an implicit short-term voice training could affect perceptual discrimination of voice cues (*f*_*o*_*+vtl*), measured in sensitivity and listening effort, with or without vocoder degradations.

**Design:**

Voice training was provided via listening to a recording of a book segment for approximately 30 min, and answering text-related questions, to ensure engagement. Just-noticeable differences (JNDs) for *f*_*o*_*+vtl* were measured with an odd-one-out task implemented as a 3-alternative forced-choice adaptive paradigm, while simultaneously collecting pupil data. The reference voice either belonged to the trained voice or an untrained voice. Effects of voice training (trained and untrained voice), vocoding (non-vocoded and vocoded), and item variability (fixed or variable consonant-vowel triplets presented across three items) on voice cue sensitivity (*f*_*o*_*+vtl* JNDs) and listening effort (pupillometry measurements) were analyzed.

**Results:**

Results showed that voice training did not have a significant effect on voice cue discrimination. As expected, *f*_*o*_*+vtl* JNDs were significantly larger for vocoded conditions than for non-vocoded conditions and with variable item presentations than fixed item presentations. Generalized additive mixed models analysis of pupil dilation over the time course of stimulus presentation showed that pupil dilation was significantly larger during *f*_*o*_*+vtl* discrimination while listening to untrained voices compared to trained voices, but only for vocoder-degraded speech. Peak pupil dilation was significantly larger for vocoded conditions compared to non-vocoded conditions and variable items increased the pupil baseline relative to fixed items, which could suggest a higher anticipated task difficulty.

**Conclusions:**

In this study, even though short voice training did not lead to improved sensitivity to small *f*_*o*_*+vtl* voice cue differences at the discrimination threshold level, voice training still resulted in reduced listening effort for discrimination among vocoded voice cues.

## INTRODUCTION

Every individual’s voice is one-of-a-kind, and voice cues can contribute to identifying speaker characteristics and telling voices apart (Abercrombie 1982). Listening to familiar voices becomes especially important in adverse listening conditions, such as at a cocktail-party (Cherry 1953), where speech streams with differing voices need to be segregated. Here, a familiar voice benefit can be achieved for normal-hearing listeners while listening to personally familiar voices, such as that of partners or close friends, in the presence of a competing talker (Johnsrude et al. 2013; Holmes et al. 2018). It is also possible to make previously unheard voices familiar through voice exposure. This voice exposure can be in the form of explicit or implicit training with voices that have different characteristics.

The effect of explicit voice training was studied by Nygaard et al. (1994) who used an experimental design in which participants were trained to remember 10 name-voice pairings with a talker identification task, over a period of 9 days. On the 10th day, word intelligibility performance was measured. A control group of participants completed the same training and testing procedure, however, the voices they were exposed to during training were not used during testing. Hence, the control group was tested with unfamiliar voices. The results showed that when presented in noise, speech produced by these familiarized voices was found to be more intelligible than unfamiliar voices. Even though explicit knowledge of talker identity (knowing who is speaking) might contribute to establishing voice familiarity, the authors argued that for listeners to show better performance on a task that did not involve explicit knowledge of talker identity, listeners should have acquired implicit talker-specific voice information during training. This talker-specific voice information was suggested to contribute to improved intelligibility when novel words were spoken by familiar voices compared to the performance shown by the control group. Hence, to some degree, these findings imply that implicit knowledge of the voices contributed to better-spoken word identification (Nygaard et al. 1994; Nygaard & Pisoni 1998).

To investigate the effects of implicit voice training, studies have implemented voice training by only exposing participants to voices, without providing explicit knowledge of talker identity (Yonan & Sommers 2000; Kreitewolf et al. 2017). Yonan and Sommers (2000) found that the talker familiarity benefit on word recognition performance, observed after either implicit or explicit voice training, was similar. Based on these findings, the authors suggested that implicit knowledge of voice characteristics, independent of explicit talker identity knowledge, plays a role in voice familiarity effects. More recently, Kreitewolf et al. (2017) implemented implicit voice training with a word identification task in speech-shaped noise, presented at different signal-to-noise ratios, for four consecutive days. The authors showed lower (better) speech-reception thresholds when speech was produced by the implicitly trained (familiar) voice compared to unfamiliar novel voices. Together, the above-mentioned studies suggest that implicitly learned aspects of voices form an important part of making them familiar.

The implicit knowledge we acquire about voices through exposure might be related to talker-specific voice cues. Two main voice cues are fundamental frequency (*f*_*o*_) and vocal-tract length (*vtl*). While *f*_*o*_ is related to the glottal pulse rate, and it is perceived as the voice pitch, *vtl* is related to speaker size and formant frequencies (Fitch & Giedd 1999). The perception of *f*_*o*_ and *vtl* is related to speaker identification (Gaudrain et al. 2009), and *f*_*o*_ and *vtl* voice cues can be used effectively by normal-hearing listeners while segregating simultaneous speech signals in speech-on-speech situations. More specifically, *f*_*o*_ and *vtl* voice cue differences between the target and the masker voices have been shown to improve the speech intelligibility of the target talker in speech-on-speech listening tasks (Darwin et al. 2003; Vestergaard et al. 2009; Başkent & Gaudrain 2016). Even though the importance of *f*_*o*_ and *vtl* differences on speech intelligibility was shown by manipulating these voice cues individually, it has also been shown that when combined, *f*_*o*_ and *vtl* differences that simulate changes in the perceived gender of a voice, results in larger improvements in speech intelligibility than shifting *f*_*o*_ or *vtl* individually (Darwin et al. 2003).

Lavner et al. (2000) recorded vowels from individuals familiar to the normal-hearing participants and morphed their voices in terms of *f*_*o*_ and *vtl*. While listening to the original and morphed versions of the vowels, participants were asked to identify the speaker by choosing the relevant name. The results showed that talker identification accuracy was hindered when *f*_*o*_ or *vtl* were manipulated individually. However, shifts in *vtl* had a larger decremental effect on performance then shifts in *f*_*o*_. The authors argued that, on average, *vtl* information was contributing to speaker individuality more than *f*_*o*_, which suggests that *vtl* is more critical for familiar talker identification, yet, there were large interindividual variations. It is also important to note that a control group, where participants were not familiar with the voices, was not included in the study. Therefore, these results only provide information on the effects of *f*_*o*_ and *vtl* manipulations on talker identification for familiar voices.

A study by Holmes et al. (2018) used speech recorded from partners of participants. The experiment consisted of both a speech intelligibility task and an explicit talker recognition task. In both tasks, the original or *f*_*o*_ and/or *vtl* shifted sentences, recorded from their partner and from unfamiliar talkers, were used. The authors showed that familiar voices could still be identified with *f*_*o*_ shifts. However, *vtl* shifts hindered the identification performance of familiar voices, which was in line with previous findings by Lavner et al. (2000). Interestingly, even when voices were no longer explicitly recognized as familiar due to voice cue shifts, the partner’s speech was still more intelligible compared to unfamiliar voices. Together, these studies (Lavner et al. 2000; Holmes et al. 2018) show that with *vtl* shifts, recognizing familiar voices becomes more difficult compared to when *f*_*o*_ of familiar voices are shifted. This suggests that *vtl* information may be more important for accurate familiar talker recognition while recognition of familiar talkers is more robust with *f*_*o*_ changes. A more recent study also showed that acoustic similarity, related to *f*_*o*_, formants, and other peaks of acoustic energy present in a voice, is an important predictor for voice identity judgments for listeners who were both familiar with the talker’s voice and unfamiliar with the talker’s voice (Lavan et al. 2021). Interestingly, for listeners who were unfamiliar with the talker’s voice, more acoustic similarity was needed between the two voices to perceive these voices as belonging to the same identity. While the above-mentioned studies provide insights about the role of voice cue information in voice familiarity, they did not directly examine the perceptual discriminability of *f*_*o*_ and *vtl* combined voice cue changes for trained and untrained voices, especially with degraded speech signals.

A special case where speech signals are inherently degraded is electric hearing via cochlear implants (for a review, see Başkent et al. 2016b). Voice characteristics such as *f*_*o*_ and *vtl* are not optimally perceived by cochlear implant users, likely contributing to difficulties in understanding speech in multi-talker situations. Studies showed that implant users have challenges with voice gender identification (Fu et al. 2004, 2005; Fuller et al. 2014) and speech intelligibility in speech-on-speech listening situations (El Boghdady et al. 2019). Sensitivity to perceptual differences in voice cues with degraded speech was studied directly by assessing just-noticeable differences (JNDs; Gaudrain & Başkent 2015, 2018). The authors used vocoders to simulate some aspects of implant processing and obtained JNDs from adult normal-hearing listeners (Gaudrain & Başkent 2015), and later measured JNDs from adult implant users (Gaudrain & Başkent 2018). Perception of both voice cues was found to be limited with vocoder manipulations and for actual implant users, and authors showed that with increased spectral degradation in the speech signal, *vtl* JNDs became larger (worse), more so than *f*_*o*_ JNDs, implying that perception of *vtl* was specifically affected by spectral degradations while *f*_*o*_ perception was more robust compared to *vtl*. The implication that for normal-hearing listeners, speech intelligibility might improve with voice familiarity in speech-on-speech situations where voice information is necessary for target and masker segregation, leads to the question of whether reduced sensitivity to *f*_*o*_*+vtl* voice cues, by vocoding, would also improve with voice training.

In addition to speech signal degradation, acoustic/linguistic variability between stimuli can affect voice discrimination performance. Children with implants were better at talker discrimination when each sentence uttered by the two talkers was the same, compared to when the linguistic content of each sentence was different (Cleary & Pisoni 2002). Koelewijn et al. (2021a) studied the effect of acoustic/linguistic variability on *f*_*o*_ and *vtl* voice cue discriminability in unprocessed and noise-vocoded speech. The authors showed that when three acoustic/linguistically different (variable) words were presented in a voice discrimination task, the *f*_*o*_ and *vtl* JNDs were larger compared to when the same word was presented three times (fixed), both for vocoded and non-vocoded speech. However, it remains unclear whether the interaction between acoustic/linguistic variability and voice cue perception in non-vocoded and vocoded speech is affected by voice training. Additionally, because voice familiarity shows the most benefit on speech perception when listening situations are not optimal, due to background noise or competing talkers (Nygaard & Pisoni 1998; Yonan & Sommers 2000; Souza et al. 2013), voice cue discrimination performance might improve for trained voices when acoustic/linguistically variable items are presented, compared to fixed items.

### Listening Effort

Listening can be challenging and effortful for normal-hearing listeners during adverse conditions such as listening in poor acoustic environments, listening to accented speech (Mattys et al. 2012; Van Engen & Peelle 2014), listening in the presence of background noise (Zekveld et al. 2010), or listening to an interfering talker (Koelewijn et al. 2012). The Framework for Understanding Effortful Listening (FUEL) (Pichora-Fuller et al. 2016) describes the listening effort as “the deliberate allocation of mental resources to overcome obstacles in goal pursuit when carrying out a listening task.” FUEL shows that demands, motivation, and attention can influence effort independently or interactively. An increase in listening effort would be beneficial during speech perception to achieve better intelligibility levels. However, when sustained for prolonged periods, it can also lead to fatigue (Pichora-Fuller et al. 2016). Hence, an ideal listening situation would be with high performance combined with low listening effort. Since performance measures do not always capture listening effort, the latter is often measured with other methods than intelligibility. In dual-task paradigms, a secondary task is conducted in parallel to the primary task of speech intelligibility (Kahneman 1973; Gagné et al. 2017), and in pupillometry measures, changes in pupil diameter over time in response to a task, do reflect changes in cognitive demand and hence cognitive load (Kahneman & Beatty 1966). More specifically, a reduction in secondary task performance, often measured in response times, or an increase in the pupil dilation response are considered to reflect an increase in listening effort.

Hearing impairment, by degrading the incoming acoustic signal, can make listening more effortful compared to normal hearing, leading to more stress, fatigue and occupational difficulties, and overall decrements in quality of life (Hétu et al. 1988; Kramer et al. 1997, 2006; Hicks & Tharpe 2002; Ohlenforst et al. 2017). Research shows that as the spectral degradation in the speech signal increases, such as can happen in implants, listening becomes more effortful (Pals et al. 2013; Winn et al. 2015; Peelle 2018), which would make adverse listening conditions even more challenging for implant users. Interestingly, Winn et al. (2015) showed that even when intelligibility was at 100%, with progressive degradation of the speech signal by means of vocoding, an increase in listening effort was observed. These findings imply that even perfect intelligibility can be coupled with a cost of increased listening effort. Based on these observations, in addition to improving voice discrimination sensitivity, trained voices may also affect cognitive processing load, especially while listening to vocoder-degraded speech.

To our knowledge, there are only a few studies addressing how voice exposure might affect listening effort (Kuchinsky et al. 2014; Koelewijn et al. 2015; Sommers et al. 2015; McKenzie et al. 2021). Koelewijn et al. (2015) examined the effect of talker uncertainty on speech intelligibility and listening effort. Pupillometry was used to assess cognitive effort while each participant listened to a target sentence either uttered by the same talker during a block or by one out of four different, randomly assigned talkers. Although the authors did not implement voice training, it was assumed that their design provided information to listeners on who was going to talk. While the target-talker uncertainty manipulation influenced neither speech intelligibility nor event-related pupil dilation, baseline pupil dilation was significantly smaller for the random-talker compared to the same-talker condition. These findings suggest that talker uncertainty can influence pupil dilation even in the absence of a performance change. Since the pupil baseline and pupil dilation responses are interrelated, this implies that talker uncertainty may affect listening effort.

There is some evidence showing that speech-perception training might lead to an improvement in word identification performance and a larger pupil dilation response (Kuchinsky et al. 2014), reflecting a higher cognitive processing load following speech-perception training. The training, as implemented by Kuchinsky et al. (2014), consisted of listening to the 400 most frequently used English words from 4 different talkers in the presence of background noise. The authors argued that training resulted in increased arousal and attention, and a more rapid discrimination of speech from noise. Since our study was not on speech intelligibility, but instead on voice identification, we can only infer the relevance of their findings to the current study. Even though the authors were not interested in investigating talker and voice familiarity per se, with repeated exposure to each voice (1600 words per voice), listeners might have acquired implicit voice knowledge to perform the task. Kuchinsky et al. (2014) show that with increased cognitive effort, word identification performance improves after voice exposure. The question remains if voice exposure could also lead to an improvement in performance without an increase in listening effort.

Related to that, there is evidence to support that speech-perception training might actually reduce listening effort. Sommers et al. (2015) implemented speech-perception training and presented all stimuli with a four-talker babble in the background to hearing-impaired listeners. Sommers et al. (2015) addressed listening effort as a behavioral measure of memory performance with a version of an n-back task. The authors argued that the implemented speech-perception training reduced cognitive effort to speech perception, with participants who received training remembering additional items in the three-back position. Even though the above-mentioned studies (Kuchinsky et al. 2014; Koelewijn et al. 2015; Sommers et al. 2015) give insights about changes in listening effort with voice exposure, these studies were not designed to study voice training per se.

A more recent study found that when an audiobook was narrated from a trained voice, errors in a driving dual-task were reduced, compared to an audiobook being narrated from an unfamiliar voice (McKenzie et al. 2021). The authors suggest that listening effort was reduced with voice training, which led to spare cognitive resources to be allocated to another task, such as driving, which contributed to driving performance. As cognitive resources are limited (Kahneman 1973), reducing listening effort would allow spare cognitive resources to be used for other cognitive processes, which can be especially helpful for patient populations with elevated levels of listening effort, such as cochlear implant users. Therefore, it is important to examine how voice training affects listening effort, especially when speech is vocoded.

### Current Study

In the present study, we investigated the effect of a short-term implicit voice training on *f*_*o*_*+vtl* voice cue discrimination, measured in sensitivity and listening effort, and with or without vocoder degradations. We hypothesized that voice training would improve sensitivity to voice cues, resulting in smaller JNDs for the trained voice compared to when listening to the untrained voice. While previous studies on voice familiarity and voice training mainly focused on speech comprehension, there are not many studies addressing how the perception of acoustic cues changes with voice familiarity, even though *f*_*o*_*+vtl* discrimination is also closely related to speech comprehension in speech-on-speech situations. Since familiar voices are more intelligible than unfamiliar voices when there is background noise (Nygaard & Pisoni 1998; Yonan & Sommers 2000; Souza et al. 2013) or background talkers (Holmes & Johnsrude 2020), we argue that one underlying reason might be that voice exposure improves voice cue sensitivity that likely leads to better segregation between speech signals. Therefore, in our study, we aimed to see if implicit voice training, through voice exposure, increases sensitivity to *f*_*o*_*+vtl* voice cues. We anticipated a decrease in voice cue sensitivity (larger JNDs) with vocoded signals compared to non-vocoded signals. We also expected a decrease in sensitivity to voice cues (larger JNDs) with increased acoustic/linguistic variability (variable items) as it would be more difficult to detect voice cue changes, than when acoustic/linguistic variability is controlled for (fixed item). This hypothesis is in line with Koelewijn et al. (2021a) who argue that acoustic/linguistically similar fixed items aid the three-alternative forced-choice (3AFC) task, whereas with variable items the 3AFC task might be more challenging as there will not be a direct acoustic reference where *f*_*o*_ and *vtl* voice cues can be extracted. We expected an interaction with voice training and vocoder conditions and an interaction with voice training and linguistic variability conditions. Research suggests that, in less favorable listening environments and more difficult conditions such as when a loud background noise (Nygaard & Pisoni 1998; Yonan & Sommers 2000; Souza et al. 2013) or a competing talker is present (Holmes & Johnsrude 2020), voice familiarity benefit on speech perception would be more apparent. Therefore, we anticipated that trained voices would improve sensitivity to voice cues especially when speech was vocoded and when acoustic/linguistically variable items were presented.

In addition to the predictions on JND scores, we hypothesized that voice training can affect listening effort, and we expected to see this via changes in the pupil responses. Previous research shows that speech-perception training can lead to a benefit in performance at the cost of an increase in cognitive effort (Kuchinsky et al. 2014), or voice training can show a benefit of reducing listening effort, without a performance improvement (McKenzie et al. 2021). Alternatively, speech-perception training can also improve performance and reduce listening effort (Sommers et al. 2015). Therefore, an increase in listening effort might be beneficial when there is a performance improvement. Similarly, there is also a benefit of voice training when there is no change in performance but there is a reduction in listening effort. What is important to point out here is that changes in pupil size, either larger or smaller pupil responses, reflect changes in speech processing load following training (Kuchinsky et al. 2014; Sommers et al. 2015; McKenzie et al. 2021), and depending on the design and interpretation of these studies, either an increase or decrease in pupil size can be seen as a benefit. Finally, we also anticipated that with vocoder-degraded signals and increased linguistic variability, task difficulty would increase, voice discrimination could become more effortful and larger pupil responses should be observed. In line with our expectation that voice familiarity would show the most benefit on voice cue perception in less favorable listening environments, we expected voice training to affect listening effort especially when vocoded voices were heard during voice discrimination, compared to when non-vocoded voices were presented and when variable items were uttered compared to fixed items.

## MATERIALS AND METHODS

### Participants

Sixteen adults (self-reported gender: 11 female and 5 male) between the ages of 21 and 39 years took part in the study. The number of participants included was based on previous studies conducted in our lab on voice cue sensitivity (Gaudrain & Başkent 2015; Koelewijn et al. 2021a). According to the demographic information obtained, there were no reports of neurological disorder, speech problems, or dyslexia. All participants were native speakers of the Dutch language, had normal hearing, and normal or corrected-to-normal vision. Hearing thresholds were measured using pure-tone audiometry, and only participants with a hearing threshold at or below 20 dB hearing level measured at octave frequencies from 250 to 8000 Hz were included in the study. At the beginning of the experimental session, participants received an explanation of the study and signed a written informed consent. Ethical approval was obtained from the institute’s Ethics Committee and participants received 8 euros per hour as reimbursement, in line with the departmental guidelines.

### Stimuli and Apparatus

#### Voice Recordings

All stimuli used for voice training and voice discrimination test were uttered by a female native Dutch speaker with an average *f*_*o*_ of 225 Hz. Based on the speaker’s height (166 cm), we estimated the *vtl* size to be approximately 13.6 cm (Fitch & Giedd 1999). An NT1-A RØDE microphone with pop-shield (RØDE Microphones LLC, Silverwater, Australia), Audient Evo 4 USB audio interface (Audient Ltd., Hampshire, United Kingdom), and Adobe Audition software (Adobe Inc., California, USA) were used for the recordings. Recordings were done at a sampling frequency of 44.1 kHz 24-bit in an anechoic room.

#### Stimuli for Voice Training

Stimuli used during the voice training task consisted of auditory recordings of the first 13 chapters of “The Twits” (de Griezels) by Roald Dahl in the Dutch language. Root-mean-square (RMS) levels were equalized among chapters using Adobe Audition 2020. The recorded female voice was processed offline in Python using World software (Morise et al. 2016) to produce the trained and untrained voices. *f*_*o*_ contour and spectral envelope were estimated and resynthesized to simulate a male voice. Manipulations on *f*_*o*_ were done by shifting the pitch contour upwards or downwards in semitones (st) that correspond to one 12th of an octave. With *vtl* shifts, the spectral envelope was either expanded or compressed shifting the location of formant frequencies, which leads to changes in the perception of a talker’s size (Smith & Patterson 2005). These *vtl* shifts, in st, reflect ratios. 1 st is a ratio of 2^(1/12), which is approximately 1.059, which corresponds to an increase of 5.9% in *vtl*. Furthermore, we manipulated *f*_*o*_ and *vtl* in opposite directions to simulate a male voice, as the male voice is lower in pitch compared to a female voice and the *vtl* is longer in males compared to females. Changes of 12 st *f*_*o*_ and either 3.6 or 3.8 st *vtl* represent the average difference in men and women voices for gender categorization (Fuller et al. 2014). In the current study, the male voice was defined as having a *vtl* of 3.8 st which was longer than of the original female voice, and a *f*_*o*_ that was half the frequency (−12 st) of the original female voice (based on Gaudrain & Başkent 2015, 2018). The original female voice was also processed, by keeping the original *f*_*o*_ and *vtl* parameters unchanged, to keep possible sound processing artifacts constant across all stimuli.

We chose book chapters as voice training materials since, with sentence materials, listeners can have access to a wider range of acoustical information related to a speaker’s voice and hence benefit more from the training than receiving voice training with isolated words (Nygaard & Pisoni 1998; Yonan & Sommers 2000). Additionally, listening to a book segment would be more motivating for the participants and allow us to implement an engaging implicit voice training design.

#### Stimuli for Voice Sensitivity and Listening Effort Measurements

Stimuli used during the voice discrimination task consisted of three-syllable consonant-vowels (CVCVCV). Individual CVs were spliced from the items in the Nederlandse Vereniging voor Audiologie (NVA) corpus, which consist of meaningful consonant-vowel-consonant Dutch words (Bosman & Smoorenburg 1995). The same female Dutch speaker who took part in auditory recordings of voice training materials also uttered the NVA corpus words. The individual CVs were spliced from the newly recorded NVA corpus words and the process of extracting the CVs was similar to that reported by Gaudrain and Başkent (2015, 2018). A total of 49 CVs were extracted, all sound files received a 60 ms cosine-shaped fade out to prevent abrupt offsets, and RMS levels were equalized using Adobe Audition. Spectral envelope and *f*_*o*_ contour were obtained from the extracted and processed CVs using STRAIGHT (Kawahara & Irino 2005), and later used for *f*_*o*_*+vtl* manipulation of CVs during the experimental adaptive procedure. The duration of the individual CV syllables that originally varied between 150 and 282 ms was equalized to 200 ms during the experimental adaptive procedure in MATLAB. The individual CV syllables were randomly selected for each trial to produce the triplet. There was a 50 ms silence between individual CV syllables in the triplet, and a 950 ms silence between each CV-triplet. In total, the duration of 3 CV-triplets was approximately 4 sec. Depending on the item variability condition, CV-triplets presented during 1 trial were either the same (fixed) or different (variable) for all three items.

In addition to non-vocoding presentations, stimuli were also presented in a vocoding condition. Vocoding manipulations were implemented in line with the procedure of Gaudrain and Başkent (2015) in MATLAB, using the vocoder implementation maintained by Gaudrain (2016). A 12-band 12th-order filter noise vocoder with cutoff frequencies between 150 and 7000 Hz was used, as these parameters produce voice identification sensitivity performance matching that of highly performing implant users. A noise vocoder was used with a set of 12th-order zero-phase bandpass filters between 150 and 7000 Hz, using the Greenwood function partitioning for cutoff frequencies of the filters (Greenwood 1990). The temporal envelope was extracted with half-wave rectification followed with low-pass filtering in each frequency band with a cutoff frequency of 300 Hz. Extracted envelopes were used to modulate a white noise carrier signal and later filtered by synthesis filters. Synthesis filters used the same settings as the analysis filters. The modulated output from all frequency bands was summed to obtain the vocoded signal, which was RMS equalized to the non-vocoded version of the signal.

#### Apparatus

During the experiment, visual task instructions and feedback were presented on a Philips Brilliance 240B screen. Auditory stimuli were presented using an Audient Evo 4 audio interface connected to Sennheiser HD 280 Pro (Sennheiser GmbH & Co., Wedemark, Germany) headphones. Sound levels of the stimuli, presented through these headphones, were calibrated to 60 dB SPL using a KEMAR Head & Torso simulator (GRAS Sound & Vibration, Holte, Denmark) together with a sound level meter (Svantek, SVAN 979, Warsaw, Poland). Calibration was performed on one side of the headphones, while a similar sound level on the other side was confirmed. All sound level calibrations were implemented in MATLAB. A MacBook 13-inch computer (Apple Inc., California, USA) and MATLAB software were used to run the experiment. To measure pupil size, a Tobii Pro Fusion eye-tracker was used (Tobii Pro AB, Tobii Technology, Stockholm, Sweden). The eye-tracker was attached to the monitor and placed centered below the screen. Two LED light panels (Ledgo E268CII Bi-color) were used, and placed on stands, one left and one right, above the participants. The light panels were standing 145 cm above the ground facing down on the participant. The brightness of the lights was adjusted during the light calibration procedure, while the light color was always kept constant at white light (daylight) at all times. Participants were sitting within the optimal 50 to 80 cm operating distance from the eye-tracker that was mounted below the screen. Their position was monitored during the experiment using the position guide from Tobii Pro Eye-Tracker Manager software.

### Procedure

During the voice training task, all participants listened to the first 13 chapters of “The Twits.” Half of the participants listened to the story uttered by the original female voice whereas the other half listened to the story uttered by the simulated male voice. A question was presented at the end of each chapter, to keep participants engaged during listening (Brennan et al. 2012). Questions were only related to the content of the story and participants did not receive any instructions to attend to the talker’s voice. Each question had one correct answer that could be selected out of four multiple-choice options without time constraints. Thus, the implemented voice training was implicit, and in total lasted approximately 30 min, as recently it was shown that listening to trained voices improves speech intelligibility even after 10 min of voice training (Holmes et al. 2021). Voice training was followed by a pupillometry set-up. For each participant, the light intensity levels of the LED panels were adjusted so that pupil size was close to the middle of its dilation range. This range was measured by recording the pupil diameter of the left eye in maximum and minimum illumination conditions and the middle of the dilation range was calculated by averaging over these outcomes. Following, the brightness of the light panels was adjusted so that the pupil size was approximately corresponding to the middle of its dilation range. In addition to the light level adjustment, each participant also performed a 5-dot location calibration, using the Tobii Pro Eye-Tracker Manager software.

**Fig. 1.**
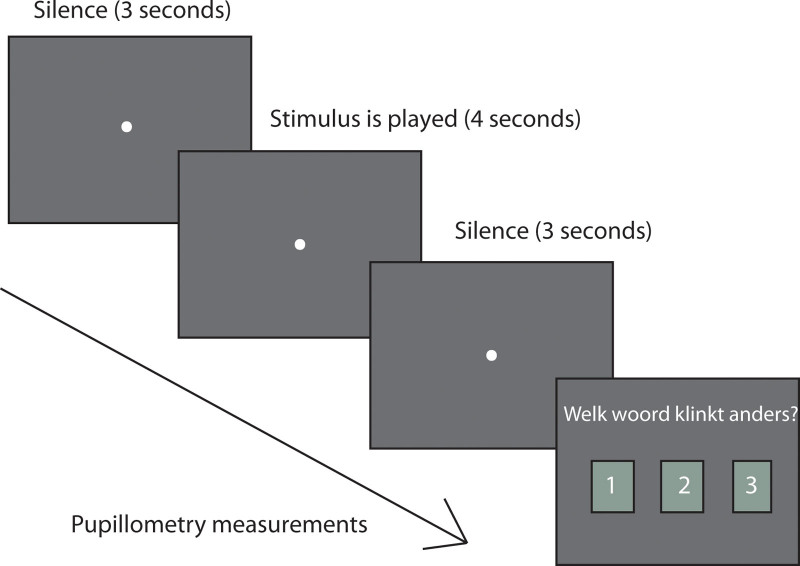
Illustration of an experimental run. The time period where pupillometry measurements were recorded is illustrated with an arrow, starting from the 3 sec of silence before the stimulus presentation and terminating at the end of the 3 sec of silence after the stimulus presentation. Stimuli were presented for approximately 4 sec. Selection boxes numbered 1 to 3 were displayed until the response was given.

During the voice discrimination task, in line with Gaudrain and Başkent (2015, 2018), JND measurements were obtained using a 3AFC task during which participants were asked to select the CV-triplet that sounded different (the target voice) than the other two identical CV-triplets (the reference voice). Before the first test run, participants were familiarized with the 3AFC task by performing a practice session containing the first 3 trials from each condition, resulting in a total of 24 practice trials. The practice session was not included in the data analysis. Each trial started with a 3-sec silence, followed by the three CV-triplets, and again followed by a 3-sec silence (Fig. 1). From the start of the 3-sec silence at the beginning of a trial, until the end of the 3-sec silence following the stimulus presentation, participants were instructed to look at a fixation dot that was presented in the center of the screen while the pupil diameters and the x- and y-gaze were recorded for both eyes. Following the 3 sec of silence after listening, selection boxes numbered 1 to 3, referring to the order of the three CV-triplets presented, appeared on the screen. Visual feedback was given to participants after their selection, with green flickering light, indicating a correct response, and an orange flickering light, in the correct location, indicating an incorrect response.

The JNDs were measured in two directions: from a simulated target male voice towards a reference female voice and from a target female voice towards a simulated reference male voice. For all participants, in half of the adaptive runs, the trained voice (female or simulated male voice) was used as a reference, while for the other half an untrained voice was used (vice versa). Reference voices were kept constant within each adaptive run. In all JND measurement directions, the target voice became closer to the reference voice by changing the *f*_*o*_ and *vtl* voice cue parameters together (*f*_*o*_*+vlt*), using STRAIGHT software (Kawahara & Irino 2005). The voice difference at the beginning of each adaptive run was 12 st and the target voice became closer to the reference voice in steps of 2 st by a 2-down-1-up staircase procedure, which corresponds to 70.7% correct on the psychometric function (Levitt 1971). The 2 st step size was updated after 15 trials or after the voice difference became smaller than twice the step size, through dividing the step size by √2. In line with the procedure of Gaudrain and Başkent (2015, 2018), we divided the step size by √2 to prevent negative differences in step sizes. This division results in logarithmic changes of step sizes and √2 are simply 1/2 on a log-scale. This approximately makes a half reduction towards dividing the step size by 2 in a logarithmic scale. Regardless of whether the trained voice belonged to the original female voice or the simulated male voice, all data was processed as either belonging to the “trained” or “untrained” voice. Participants completed JND test runs with variable or fixed items, presented in vocoded or non-vocoded conditions, with a trained or an untrained voice as the reference voice, resulting in a total of eight adaptive runs, one for each of the eight conditions. Even though it is common to include several adaptive runs per condition, we included one adaptive run per condition to keep the timing of the experiment constrained for pupillometry measurements. That way we aimed to avoid participants getting overly tired during the experiment which could affect the pupil response. The order of JND runs was randomized and each run ended after 12 reversals or 150 trials and the JNDs were calculated by averaging over the last 10 reversals. In case the maximum number of trials was exceeded, or the voice difference became too large because all the last 15 trials or more were incorrect, the same experimental condition would be repeated. Repetition of experimental conditions was introduced as a precaution, especially for situations where JNDs are larger such as in cochlear implant users. In our study, none of our participants needed to repeat any experimental condition. Testing was done in a quiet sound-treated room and simultaneous measurements of voice sensitivity and pupillometry lasted one and a half hours. In total, the experimental procedure, including pure-tone audiometry, voice training, and voice discrimination tasks, lasted approximately two and a half hours.

### Data Analysis

#### Voice Sensitivity Data Analysis

Voice sensitivity data analysis was in line with Gaudrain and Başkent (2015). Participants’ *f*_*o*_*+vtl* JNDs were analyzed with R (v4.0.2; R Core Team 2020), using the ez package (v4.4.0; Lawrence 2016). JNDs were log-transformed to reduce skewness and obtain a more normal distribution. We carried out a 2 × 2 × 2 repeated-measure analysis of variance (ANOVA) with voice training (untrained, trained voice), vocoder (non-vocoded, vocoded), and item variability (fixed, variable) as within-subject factors, on the log-transformed JNDs. Generalized eta-squared measures are reported as effect sizes (Bakeman 2005). Multiple comparisons were conducted as a pairwise *t*-test using afex (v0.28.1; Henrik et al. 2021) and lsmeans (v2.30.0; Lenth 2018) packages and type I errors were corrected with false discovery rate (FDR).

#### Listening Effort Data Analysis

Pupil data preprocessing was in line with Koelewijn et al. (2021b) and was performed in MATLAB. Pupil diameter and x- and y-gaze data were recorded for both eyes at a sample rate of 120 Hz. For each participant, the data of the eye that was of the highest quality (most valid data points due to better eye-tracking) was selected to be used for the analysis. Eye-blinks were defined as zero values in the pupil diameter traces within 1 sec before and 6.5 sec after the beginning of stimuli presentation and trials with 20% or more blinks (3.96% of trials) were excluded from further analysis. On the remaining pupil diameter and x- and y-gaze traces, blinks were removed by means of linear interpolation. Linear interpolation was conducted between 10 samples before and 16 samples after the blink. Trials where eye movements (saccades) were outside the visual display screen area (3.91% of trials) were also removed from further analysis. To the remainder of pupil traces, an 11-point moving average smoothing filter was applied to remove high-frequency artifacts. For each of the remaining traces, a baseline value was calculated by taking the mean of the 1 sec time window before stimulus onset. All of these traces were baseline corrected by subtracting the trace’s baseline value from the value for each time point within that trace. The baseline corrected pupil traces were averaged for each condition and for each participant and peak pupil dilation (PPD), mean pupil dilation (MPD), peak pupil dilation latency (PPDL), and averaged baseline pupil diameter values were calculated accordingly. PPD, MPD, and PPDL reflect event-related responses. Within the time window from stimulus onset till 6.5 sec after, which was 0.5 sec before selection boxes appeared on the screen, MPD was calculated as the average pupil dilation in mm relative to the baseline. With MPD, the mean cognitive load spent during different conditions can be compared. Within this same time window, PPD was calculated as the maximum pupil dilation value in mm compared to baseline and PPDL was calculated as the time (ms) from the onset of the stimulus until the PPD. PPD reflects the maximum cognitive load spent, related to the listening task, and PPDL reflects the time spent to reach the maximum cognitive load, relative to the start of the auditory stimuli. The baseline, which is not event-related, reflects the level of arousal before the start of the auditory stimuli. PPD, MPD, PPDL, and baseline are outcome measures from different parts of the response curves (max amplitude, the latency of maximum amplitude, etc.), and are more traditional pupil response measures.

First, we performed a 2 × 2 × 2 repeated measures ANOVA with voice training (untrained, trained voice), vocoder (non-vocoded, vocoded), and item variability (fixed, variable) as within- subject factors on the dependent variables PPD, MPD, PPDL, and pupil baseline separately, using Python and the statsmodels package (v0.12.2; Seabold & Perktold 2010). In addition, a non-linear regression analysis was performed using Generalized Additive Mixed Models (GAMMs) to assess the change in pupil dilation over time (Van Rij et al. 2019). Compared to the more traditional approaches, with GAMM, pupil dilation to different conditions can be compared over the time course by including non-linearity. Hence it is a powerful tool to spot any differences between two whole curve responses and catch differences along the curves that may or may not happen at max amplitude.

We performed the GAMM analysis on R, using the mgcv (v1.8-31; Wood 2017) and itsadug (v2.4; Van Rij et al. 2020) packages. We first pooled data over item variability conditions, as item variability had no effect on the pupil dilation and performed the GAMM analysis as a planned comparison between vocoder and voice training conditions. Before conducting the GAMM analysis, we applied eye-movement correction and de-blinking the same way as described above, and we down-sampled the data from 120 Hz to 30 Hz. We conducted a GAMM analysis with pupil diameter (mm) over time as a dependent variable with 4 conditions, looking at contrasts between the trained and untrained voices for the non-vocoded and vocoded conditions separately, and contrasts between non-vocoded and vocoded conditions for trained and untrained voices.

## RESULTS

### Voice Training

Thirteen multiple-choice questions were presented at the end of each story chapter to keep participants engaged during the task. The overall accuracy score of participants was high (M = 88.94%, SD = 9.45%) and no participant scored lower than 76.92% correct. The high accuracy score confirmed that participants were attentive during the story-listening task.

### Voice Sensitivity

**Fig. 2.**
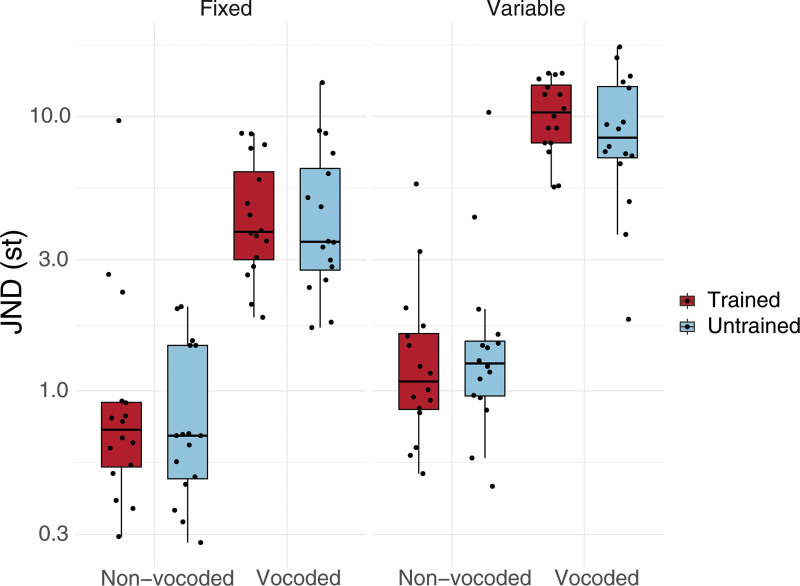
Sensitivity to voice cues, shown in *f_o_+vtl* JNDs for voice training (untrained, trained), item variability (fixed, variable), and vocoder (non-vocoded, vocoded) conditions. JNDs on the y axis are plotted on a logarithmic scale. The boxes illustrate the lower and upper quartiles, and the midline of the boxes denotes the median. The whiskers show the highest and lowest values within 1.5 times the interquartile range from above the upper and below the lower quartiles, respectively. The black dots represent data points from individual participants for each condition. JNDs indicate just-noticeable differences.

Figure 2 shows the *f*_*o*_*+vtl* JNDs for different voice training, vocoder, and item variability conditions, and Table 1 shows the average *f*_*o*_*+vtl* JNDs in semitones for each experimental condition. Table 2 shows results from the 2 × 2 × 2 repeated measures ANOVA on the JNDs with voice training (untrained, trained), vocoder (non-vocoded, vocoded), and item variability (fixed, variable) as within-subject factors.

**TABLE 1.** Averaged *f_o_+vtl* JNDs shown in semitones (st) for each condition and their SD

Item Variability	Vocoder	Voice Training	JNDs (st)	SD
Variable	Non-vocoded	Trained	1.52	1.30
		Untrained	1.95	2.40
	Vocoded	Trained	10.41	3.03
		Untrained	9.31	4.48
Fixed	Non-vocoded	Trained	1.43	2.29
		Untrained	0.90	0.59
	Vocoded	Trained	4.70	2.33
		Untrained	4.91	3.20

JNDs, just-noticeable differences.

published online ahead of print January 25, 2023.

**TABLE 2.** Results from repeated measures ANOVA

*f_o_+vtl*	*F*	*p*	*η^2^_g_*
Voice training (VT)	1.16_[1,15]_	0.298	<0.01
Vocoder	346.74_[1,15]_	**<0.001**	0.69
Item variability (IV)	65.03_[1,15]_	**<0.001**	0.21
VT × vocoder	0.42_[1,15]_	0.528	<0.01
VT × IV	0.12_[1,15]_	0.736	<0.01
Vocoder × IV	8.82_[1,15]_	**0.010**	0.01
VT × Vocoder × IV	2.67_[1,15]_	0.123	<0.01

Effects of voice training (untrained, trained), vocoder (non-vocoded, vocoded), and item variability (fixed, variable) on *f_o_+vtl* JNDs.

*η^2^_g_*, generalized eta squared; JNDs, just-noticeable differences.

Boldface indicates significant results.

The results indicate that vocoder had the largest significant main effect on the JNDs (*F*_(1,15)_ = 346.74, *p* < 0.001, *η^2^_g_* = 0.69), with vocoded conditions showing on average larger JNDs (7.33 st), compared to non-vocoded conditions (1.45 st). Item variability had the second largest significant main effect on the JNDs (*F*_(1,15)_ = 65.03, *p* < 0.001, *η^2^_g_* = 0.21). When averaged over all conditions, variable item presentations yielded larger JNDs (5.80 st) compared to when fixed (same) items were presented (2.99 st). Voice training did not have a significant main effect on the JNDs (*F*_(1,15)_ = 1.16, *p* = 0.298, *η^2^_g_* = 0.00); when averaged across conditions, the average JNDs for untrained voices were 4.27 st and average JNDs for trained voices were 4.52 st.

A significant interaction was observed between vocoder and item variability conditions on the JNDs (*F*_(1,15)_ = 8.82, *p* < 0.05, *η^2^_g_* = 0.01), although the effect size was relatively small. Multiple comparisons were conducted as a pairwise *t*-test between the vocoder and item variability conditions. Item variability conditions (variable vs fixed) differed significantly from each other in non-vocoded conditions (*t*_(15)_ = 5.38, *p*_*FDR*_ < 0.001), as well as in vocoded conditions (*t*_(15)_ = 8.43, *p*_*FDR*_ < 0.001). JNDs were larger when vocoded variable items were presented (9.86 st) compared to vocoded fixed items (4.81 st), and when non-vocoded variable items were presented (1.74 st) compared to non-vocoded fixed items (1.17 st), indicating larger JNDs in both vocoded and non-vocoded conditions with variable items than fixed items and larger JNDs with vocoder conditions in general. There were no significant interactions between voice training and vocoder (*F*_(1,15)_ = 0.42, *p* = 0.528, *η^2^_g_* = 0.00) and no significant interactions between voice training and item variability (*F*_(1,15)_ = 0.12, *p* = 0.736, *η^2^_g_* = 0.00). Finally, there was no 3-way interaction among voice training, vocoder, and item variability (*F*_(1,15)_ = 2.67, *p* = 0.123, *η^2^_g_* = 0.00).

### Listening Effort

**Fig. 3.**
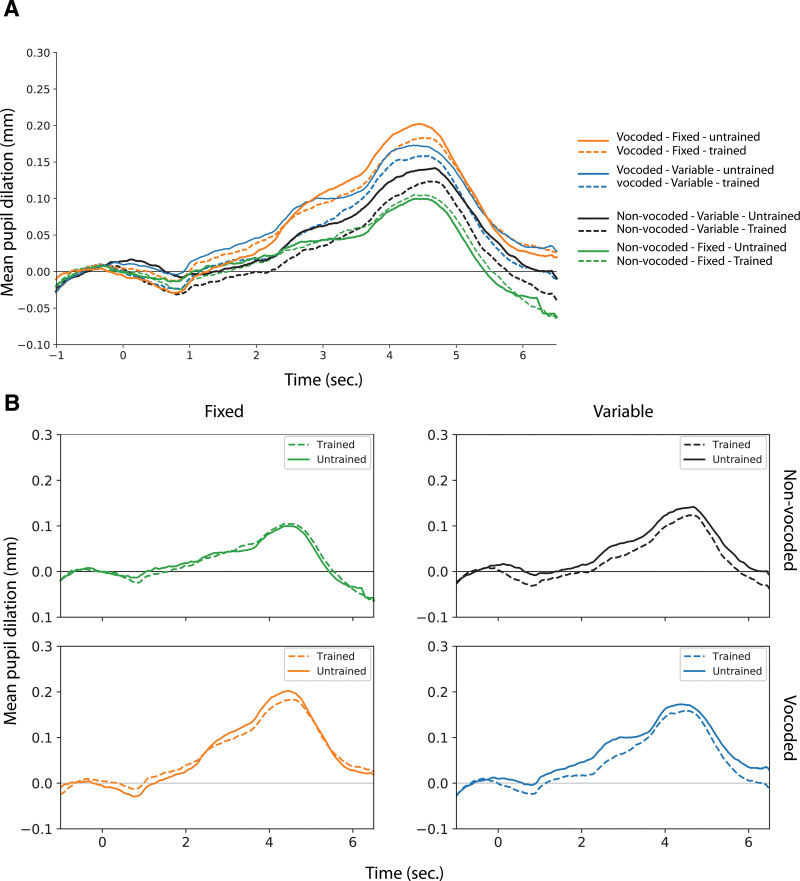
Listening effort is shown in pupil dilation (mm) over time. The time between −1 and 0 sec is considered the baseline period and from 0 to 6.5 sec represents the time period where the stimuli are presented, and pupil data are recorded and analyzed. Dashed and solid lines illustrate the conditions with trained and untrained voices, respectively. A, Mean pupil dilation over time illustrated with all conditions. B, Mean pupil dilation over time for trained vs untrained voices plotted separately for the vocoder and item variability conditions.

Figure 3A shows the pupil dilation (mm) relative to baseline over time with pupil traces averaged over participants for each condition. The pupil dilation while listening to trained (dashed lines) compared to untrained (solid lines) voices are shown as separate plots for the corresponding vocoder and item variability conditions in Figure 3B. Table 3 shows the results from four different 2 × 2 × 2 repeated measures ANOVA on PPD, MPD, PPDL, and baseline.

**TABLE 3.** Results from 4 different repeated measures ANOVA

Main Effects	Voice Training (VT)				Vocoder		Item Variability (IV)	
	*F*		*p*		*F*	*p*	*F*	*p*
PPD	2.38		0.144		14.82	**0.002**	0.00	0.976
MPD	1.31		0.270		10.90	**0.005**	0.06	0.812
PPDL	0.72		0.409		7.50	**0.015**	0.01	0.945
Baseline	0.11		0.740		0.00	0.957	12.67	**0.003**
Interactions	VT × Vocoder		VT × IV		Vocoder × IV		VT × Vocoder × IV	
	*F*	*p*	*F*	*p*	*F*	*p*	*F*	*p*
PPD	0.03	0.867	0.10	0.758	1.65	0.218	0.15	0.708
MPD	0.00	0.970	1.05	0.321	0.76	0.398	0.00	0.968
PPDL	0.35	0.565	0.18	0.679	0.27	0.611	0.08	0.787
Baseline	1.71	0.211	0.06	0.805	4.88	**0.043**	1.18	0.294

Effects of voice training (VT), vocoder, and item variability (IV) on peak pupil dilation (PPD), mean pupil dilation (MPD), peak pupil dilation latency (PPDL), and baseline.

Boldface indicates significant results.

As shown in Figure 3, vocoded conditions yield larger pupil dilation responses, compared to non-vocoded conditions. Results from ANOVA further indicate a significant main effect of vocoder on PPD (*F*_(1,15)_ = 14.82, *p* < 0.01), MPD (*F*_(1, 15)_ = 10.90, *p* = <0.01), and on PPDL (*F*_(1, 15)_ = 7.50, *p* < 0.05). Even though untrained voices seem to yield larger pupil responses compared to trained voices in all conditions except for non-vocoded condition with fixed items (Fig. 3B), there was no significant main effect of voice training on PPD (*F*_(1, 15)_ = 2.38, *p* = 0.144), MPD (*F*_(1, 15)_ = 1.31, *p* = 0.270), or PPDL (*F*_(1, 15)_ = 0.72, *p* = 0.409). Finally, there was no significant main effect of item variability on PPD (*F*_(1, 15)_ = 0.00, *p* = 0.976), MPD (*F*_(1, 15)_ = 0.06, *p* = 0.812), or PPDL (*F*_(1, 15)_ = 0.01, *p* = 0.945). Interestingly, item variability affected the baseline (*F*_(1, 15)_ = 12.67, *p* < 0.01). Pupil dilation during the baseline period was on average larger when variable items (3.07 mm) were presented compared to fixed items (3.03 mm). There was also an interaction between vocoder and item variability conditions on the baseline (*F*_(1, 15)_ = 4.88, *p* < 0.05). Multiple comparisons between vocoder and item variability conditions on the baseline showed a significant difference between variable and fixed items in vocoded conditions (*t*_(28,7)_ = 3.95, *p*_*FDR*_ < 0.001), but there was no significant difference between variable and fixed items in non-vocoded conditions (*t*_(28,7)_ = 0.51, *p*_*FDR*_ = 0.616). No other significant interactions for any of the pupil measures were observed (Table 3).

### GAMM Analysis Results

We analyzed pupil dilation response over time with GAMMs. In GAMM analysis, smooth functions are fitted to the data and the relation between variables is not forced to be linear. For details of performing GAMM analysis on pupillometry data, see Van Rij et al. (2019). We performed a GAMM analysis as a planned comparison between vocoder and voice training conditions on the pupil dilation, by pooling the item variability condition data, since there was no evidence of item variability affecting the pupil dilation, or interacting with any of the other independent variables, after baseline correction. To check if pooling item variability would affect the MPD, we performed an additional 2 × 2 ANOVA on MPD by pooling item variability. The results from this analysis were similar to the outcomes of the full model.

**Fig. 4.**
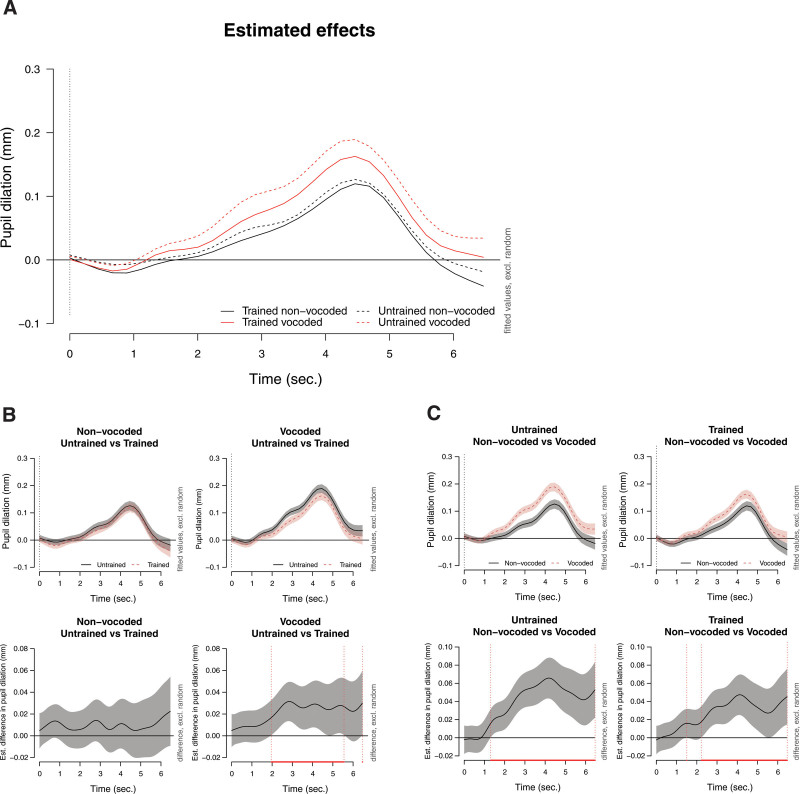
Listening effort is shown with GAMM analysis. Stimuli were presented from 0 until approximately 4 sec, followed by a silence, and no response was given before 6.5 sec. A, Estimated effects (fitted effects) of pupil dilation for trained and untrained voices in vocoded and non-vocoded conditions. B, Estimated differences in pupil dilation between untrained and trained voices within vocoded and non-vocoded conditions across time are shown, with pointwise 95% confidence intervals. The red line on the lower right panel x axis indicates the time period where the estimated difference in pupil dilation between the untrained and trained voices significantly differs from the 0 line. C, Estimated differences in pupil dilation between non-vocoded and vocoded speech across time is shown for untrained and trained voices, with pointwise 95% confidence intervals. The red line on the x axis of lower panels indicates the time period where the estimated differences in pupil dilation between the non-vocoded and vocoded speech significantly differ from the 0 line. GAMM indicates generalized additive mixed model.

The function *bam* from the mgcv package and the following syntax was used: Pupil ~ Condition + s(Time, by = Condition, k = 15) + s(Event, bs = “re”) + s(Time, Event, bs = “re”). Autocorrelation of errors was controlled with the use of the *acf_resid* function from the itsadug package and the corresponding output *rho* was added to the model. Here, the condition was defined as a factor of voice training and vocoder conditions and estimated effects were calculated for each condition on the dependent variable Pupil (Fig. 4A). Figure 4B shows the estimated differences in the pupil dilation between untrained and trained voices for non-vocoded and vocoded conditions separately. A significant deviation from the 0 line is illustrated as a red line on the below panel of estimated difference plots. A visual inspection of the estimated difference plots indicates a significant difference between the untrained and trained voices on the pupil dilation response while listening to vocoded signals. Binary difference smooth statistics confirmed that the intercept (constant)+non-linear difference between the untrained and trained voices for vocoded speech was significant (*p* < 0.01) and pupil dilations were significantly larger when speech was spoken from the untrained voice compared to trained voice for vocoded speech. Ordered factor smooth statistics were also assessed to distinguish the intercept and the non-linear differences in the data. The non-linear difference between untrained and trained voices for vocoded speech was also significant (*p* < 0.05). However, there was no significant difference between conditions of voice training on the pupil dilation while listening to non-vocoded speech signals, according to the binary smooth difference statistics (*p* = 0.54). Ordered factor statistics revealed that there was also no significant non-linear difference between untrained and trained voices for non-vocoded speech (*p* = 0.55). These results also imply that the differences between untrained and trained voices for vocoded and non-vocoded speech are better explained with both linear and non-linear patterns in our data. Figure 4C shows the contrast between non-vocoded and vocoded speech for untrained and trained voices. Binary smooth difference statistics, ordered factor difference statistics, and visual inspection of the plots indicate that pupil dilation responses were significantly different between non-vocoded and vocoded speech, when speech was spoken by an untrained voice (*p* < 0.01) and a trained voice (*p* < 0.01). According to binary smooth difference statistics, there was no significant intercept (constant) + non-linear difference between non-vocoded and vocoded speech, between trained and untrained voices (*p* = 0.57). However, this interaction was shown to be significant with ordered factor difference statistics, indicating that there was an interaction between voice training and vocoding (*p* < 0.05), which was better explained with non-linearity. The interaction indicates that estimated differences in pupil dilation responses to non-vocoded and vocoded speech were smaller when speech was spoken from a trained voice compared to an untrained voice. The deviance explained from the final model was 77.1%.

Table 4 shows the summary statistics for parametric coefficients and smooth terms. The parametric coefficients show the linear differences within the data. Significance levels of conditions are shown with *p* values, in comparison to the intercept, which is the reference condition, untrained non-vocoded. Smooth terms represent the data over time and capture non-linear patterns. The approximate significance of the smooth terms is shown by means of *p* values. The Ref.df denotes the reference number of degrees of freedom used and the edf value represents the number of effective degrees of freedom. The Edf value indicates the amount of non-linearity of the smooth (Wieling 2018).

**TABLE 4.** GAMM analysis results

Parametric Coefficients	Estimates	SE	*t*-value	Pr (> t )	
(Intercept)	0.03043	0.00675	4.51	6.6e-06	***
Condition trained vocoded	0.02627	0.00926	2.84	0.0046	**
Condition trained non-vocoded	0.00951	0.00958	0.99	0.3212	
Condition untrained vocoded	0.04735	0.00922	5.13	2.9e-07	***
Smooth terms	edf	Ref.df	*F*	*p*	
s(Time):Conditiontrainednonvocoded	12.5	13.7	171	<2e-16	***
s(Time):Conditiontrainedvocoded	13.0	13.8	244	<2e-16	***
s(Time):Conditionuntrainednonvocoded	12.5	13.7	150	<2e-16	***
s(Time):Conditionuntrainedvocoded	13.1	13.9	280	<2e-16	***
s(Event)	2932.8	3399.0	467	<2e-16	***
s(Time, Event)	3101.8	3398.0	506	<2e-16	***

Summary statistics of parametric coefficients and smooth terms for time and condition, as a factor of voice training and vocoder condition levels, are shown.

Deviance explained = 77.1%

Significance codes: *p < 0.05, **p < 0.01, ***p < 0.001.

GAMM, generalized additive mixed model.

## DISCUSSION

The goal of the current study was to investigate the effect of voice training, on *f*_*o*_*+vtl* voice cue discrimination, measured in sensitivity (quantified with JNDs) and listening effort (quantified with MPD, PPD, PPDL, and non-linear time course measurement of pupil dilation using GAMM). The speech signal was either non-vocoded or vocoded, and the amount of acoustic/linguistic information was manipulated by presenting fixed or variable items.

Voice training effects were mixed. Voice cue sensitivity results showed that, contrary to our expectations, voice training did not influence the JNDs. Also, contrary to our hypothesis, no main effect of voice training on the pupil data measured with MPD, PPD, and PPDL was observed. However, planned comparisons time course analysis (GAMM) did show an effect of voice training on the pupil size. There was a larger pupil dilation response for untrained voices relative to trained voices in the vocoded listening condition. This suggests a decrease in listening effort during voice cue discrimination for trained compared to untrained voices when, because of vocoding, input-related processing demands are high, perhaps as a result of compensatory processes for degraded speech (Davis & Johnsrude 2007; Başkent, Clarke, et al. 2016a; Başkent et al. 2016b).

Other effects related to the experiment were as predicted. JNDs were higher (poorer discrimination) with vocoder manipulations than when the speech signal was unprocessed (in line with Gaudrain & Başkent 2015). Vocoding also resulted in a clear increase in the pupil dilation response, as reflected by the MPD, PPD, and PPDL, for vocoded speech relative to non-vocoded speech (in line with Winn et al. 2015). Finally, our results showed larger JNDs when variable items were presented compared to fixed items (in line with Koelewijn et al. 2021a). Item variability only affected the pupil baseline that also showed an interaction between item variability and vocoding, which was also reflected by the JNDs.

### Voice Cue Discrimination

Even though experimental design choices were carefully made, as explained in detail in Methods, we did not find an effect of voice training on the JNDs. Our decision to use an audiobook was based on its successful use in previous research (Plant et al. 2015; Humes et al. 2019; Jiam et al. 2019; McKenzie et al. 2021), and our aim to implement an engaging, short-term, implicit voice training. Engagement was an important aspect of our study. According to the adaptive gain theory (Aston-Jones & Cohen 2005), phasic mode, related to task engagement, improves focus and task performance. Engagement also prevents distractibility, which would be evident by the pupil response during the 3-AFC task. In addition to being engaging, from the linguistic content perspective, audiobooks contain properties of continuous speech with rich context, and sentence materials provide access to a wide range of acoustic characteristics of a speaker’s voice (Nygaard & Pisoni 1998; Yonan & Sommers 2000). For implant users, audiobooks have been used for voice training and auditory training (Plant et al. 2015; Jiam et al. 2019), and pitch discrimination in implant users was shown to improve with such techniques (Jiam et al. 2019). Some studies showed a relation to voice familiarity with training, using audiobooks. When an implicit short-term voice training was implemented using an audiobook, a dual-task of driving and listening to another chapter of the audiobook showed that the number of errors in driving decreased, when the audiobook was narrated by the trained voice compared to an untrained voice (McKenzie et al. 2021). It is suggested that listening to trained voices makes listening less effortful, which allows spare cognitive resources to be allocated to another task. In turn, this resulted in better driving performance. This is now confirmed by the current pupil dilation data. Additionally, we had chosen to implement an implicit voice training instead of explicit, as implicit voice training more closely resembles the processes of becoming familiar with voices in real life. Studies showed that through both explicit voice training (Nygaard et al. 1994; Nygaard & Pisoni 1998; Levi et al. 2011) and implicit voice training (Yonan & Sommers 2000; Kreitewolf et al. 2017) speech intelligibility was improved, and the intelligibility benefit obtained from the two voice training methods was found to be similar (Yonan & Sommers 2000). In terms of training duration, even though the voice familiarity benefit might be enhanced with longer training implementations, such as training for 4 days (Kreitewolf et al. 2017) or 9 days (Nygaard et al. 1994), short-term voice training implementations, which can be 10 min (Holmes et al. 2021), 20 min (McKenzie et al. 2021), or 2 days (Case et al. 2018), have also been effectively used to establish voice familiarity. One advantage of shorter-duration training is that a larger number of people tend to complete the training (Jiam et al. 2019), which could become especially important for implant users, when training methods are used for rehabilitation purposes.

We expected that voice training would improve voice cue discrimination as previous research had shown that familiar voices are more intelligible than unfamiliar voices (Nygaard et al. 1994; Nygaard & Pisoni 1998; Yonan & Sommers 2000; Johnsrude et al. 2013; Kreitewolf et al. 2017; Holmes et al. 2018). Voice familiarity benefit on intelligibility was especially apparent when listening situations are less favorable, such as listening to a familiar talker in the presence of a competing talker (Holmes & Johnsrude 2020), or noise, when the signal-to-noise ratio was lowest (Nygaard & Pisoni 1998; Yonan & Sommers 2000; Souza et al. 2013). Previous research also showed that to identify familiar talkers, *f*_*o*_ and *vtl* voice cues are used (Lavner et al. 2000; Holmes et al. 2018). Although *f*_*o*_ and *vtl* information is used to recognize familiar talkers and understand speech spoken by familiar voices, voice manipulations used by Holmes et al. (2018) were above the threshold. More specifically, the median *f*_*o*_ and *vtl* thresholds were adaptively measured at 90% performance before the actual experiment, and then multiplied by five for the actual experiment. These findings suggest that, compared to unfamiliar voices, familiar voices may be more intelligible and recognized as familiar talkers with large *f*_*o*_ and *vtl* shifts, however, our results suggest that voice training does not improve sensitivity to small differences of *f*_*o*_*+vtl* at the 70.7% correct performance threshold level, even under difficult listening conditions, such as with vocoder degraded speech and when items were acoustic/linguistically variable.

Regardless of voice sensitivity findings, listeners most likely still implicitly learned voice information related to, but not limited to, *f*_*o*_*+vtl* from the trained voice. Even though we did not have a measure of whether voice familiarity was successfully established with the current voice training, results from content-related multiple-choice questions presented during audiobook listening showed that participants were listening attentively. Additionally, the current voice training did show a benefit in reducing listening effort. Our results may be implying that differentiation of small changes in voice cues did not depend on voice training, and voice discrimination based solely on changes in *f*_*o*_*+vtl* information, without any changes on other covarying voice cues, was not sufficient to see a voice training benefit on performance. Additionally, contrary to previous studies where voice training was followed by tests with the same tasks, (Kreitewolf et al. 2017), and tests with similar materials, such as words (Nygaard & Pisoni 1998; Kreitewolf et al. 2017) or sentences (Yonan & Sommers 2000), in the current study, the goal was to see whether voice information related to *f*_*o*_*+vtl* that was learned implicitly during voice training can be generalized and used for a task with different materials. Nygaard & Pisoni (1998) showed that when sentences were used for voice training, intelligibility test performance was better when sentences were used, compared to isolated words. Therefore, it is possible that using linguistically similar materials in training and testing might have resulted in better performance in the JND task for trained voices. Hence, voice information from training with meaningful sentences (audiobook) did not generalize to differentiating small voice cue changes of isolated meaningless CV-triplets, although the same paradigm still resulted in voice training benefits measured in listening effort.

### Vocoder and Item Variability

In line with previous studies (e.g., Gaudrain & Başkent 2015), the current results for vocoded stimuli showed that spectrotemporal degradation of the speech signal, such as with acoustic vocoder cochlear implant simulations, impaired perceptual discriminability of *f*_*o*_ and *vtl* voice cues. In their first study, Gaudrain & Başkent (2015) used two vocoder conditions and a non-vocoder condition. For vocoder manipulations, they used a sinewave vocoder with either a 12-band 12th order filter vocoder (higher spectral resolution) or with a 4-band 12th order filter (lower spectral resolution) vocoder while presenting CV-triplets for measuring *f*_*o*_ and *vtl* JNDs. In the current study, *f*_*o*_*+vtl* JNDs obtained with non-vocoded conditions were similar to the JNDs Gaudrain and Başkent (2015) reported for voice cue discriminability of non-vocoded speech.

Our results showed, as predicted, that voice discrimination was hindered (larger JNDs) when variable items were presented compared to fixed items. This finding is in line with previous studies, suggesting that acoustic/linguistic variability could interact with voice discrimination (Mann et al. 1979; Cleary & Pisoni 2002; Koelewijn et al. 2021a). One of these previous studies showed that normal-hearing children were better at voice discrimination when talkers uttered the same sentence instead of different sentences (Mann et al. 1979). In addition to normal-hearing children, Cleary and Pisoni (2002) studied talker discrimination in different linguistic variability conditions with pediatric implant users. The authors’ findings indicate that children with implants, like normal-hearing children, were also better able to discriminate between talkers when the talkers all uttered identical sentences, compared to when they each spoke a different sentence. With direct manipulation of *f*_*o*_ and *vtl* voice cues, Koelewijn et al. (2021a) studied the effect of linguistic variability in unprocessed and noise-vocoded speech, in normal-hearing adults. In line with Cleary and Pisoni (2002), they showed that *f*_*o*_ and *vtl* JNDs were larger when three acoustic/linguistically different words were presented than when the same words were presented while listening to both vocoded and non-vocoded speech. Interestingly, in the current study, listening to fixed items improved sensitivity to *f*_*o*_*+vtl* voice cue discrimination more for vocoded than for non-vocoded conditions, as shown by the significant interaction. This outcome suggests that acoustic/linguistic similarities between items not only helped normal-hearing listeners discriminate among unprocessed voices but helped listeners even more for discriminating vocoded voices where the speech signal is spectrotemporally degraded.

### Listening Effort

Against the expectations based on our hypotheses, listening to trained or untrained voices during the JND task did not significantly affect PPD and MPD outcomes. Recently, the limitations of analyzing maximum (peak) and MPD within a specific time window were discussed (Winn et al. 2015; Van Rij et al. 2019). Even though measures like PPD and MPD are used often, and can reduce the complexity of the analysis, it is suggested that task-related differences in pupil dilation can be better captured with a time course analysis and with this approach, any change over any timeframe could be detected. Therefore, to get a more complete picture of our pupil data, we analyzed the change in pupil dilation over time with GAMMs. According to our GAMM analysis results, pupil dilation was significantly larger during voice discrimination of untrained vocoded voices, compared to voice discrimination of a trained vocoded voice.

Specifically, untrained vocoded voices yielded larger pupil dilation responses compared to trained vocoded voices from 2 to 5.5 sec, which was almost the entire duration of the stimuli presentation (Fig. 4B). In line with our predictions, there was no significant difference between conditions of voice training on the pupil dilation while listening to non-vocoded speech. This is in line with previous research suggesting that listening to familiar voices shows the most benefit in less favorable listening conditions (Nygaard & Pisoni 1998; Yonan & Sommers 2000; Souza et al. 2013; Holmes & Johnsrude 2020). Therefore, it is not entirely surprising to observe that listeners benefit from voice training when speech was vocoder degraded and not when speech was unprocessed. Pupil dilation responses to non-vocoded and vocoded speech were also smaller when speech was spoken from the trained voice compared to the untrained voice (Fig. 4C). These results indicate that pupil responses were less affected by vocoding for trained voices.

Important to note is that increased listening effort while listening to untrained vocoded voices could reflect compensatory processes that counteracted a performance drop in JNDs for untrained voices relative to trained voices, which hindered the expected performance benefit from listening to trained voices. This is in line with our expectation that voice training would show the most effect on listening effort during voice cue perception in less favorable listening environments. As shown by Koelewijn et al. (2012), in specific challenging listening situations, increased listening effort could reflect a compensation mechanism that counteracts a drop in performance or even lead to improved intelligibility. The authors showed that speech-reception thresholds were slightly better when a single-talker masker was used compared to fluctuating noise, despite the fact that the pupil dilation responses were significantly larger (more effort) with single-talker masker than fluctuating noise. Additional speech content and talker (e.g., voice) information available in the single-talker masker compared to the fluctuating noise masker could have been utilized to improve target masker segregation, and in turn enhance task performance. Processing this additional masker information, and/or deploying attentional resources that can help separate the target and masker signals, might have increased the cognitive load, reflected by a larger pupil dilation response. Additionally, it is suggested that directed attention, as reflected by an increase in cognitive load, is necessary for the improvement of vocoder-degraded speech intelligibility (Wild et al. 2012). Accordingly, because listening to untrained vocoded voices resulted in a larger pupil dilation response than trained vocoded voices, this larger response could reflect the recruitment of cognitive resources that might have improved voice cue discrimination performance. Our results show that, even though not a significant difference, average JNDs for vocoded untrained voices were lower (7.11 st) than vocoded trained voices (7.56 st). Therefore, voice cue discrimination performance might have improved while listening to untrained voices and was similar to voice cue discrimination thresholds obtained while listening to trained voices. Nevertheless, this explanation only incorporates voice cue discrimination performance with vocoded voices and does not provide an explanation for performance on non-vocoded voice cue discrimination. Still, it is in line with the idea that compensation processes are likely to be effectuated during difficult listening situations with hearing impairment or otherwise degraded speech (Başkent et al. 2016b).

With the effect of vocoding on listening effort, our findings were in line with our predictions that listening to vocoded speech would elicit larger pupil responses than listening to unprocessed speech. This finding is also in line with previous studies where an increased listening effort was reported when the speech signal was degraded (Pals et al. 2013; Winn et al. 2015; Peelle 2018). Winn et al. (2015) showed that with decreased number of spectral bands on the vocoder, hence more degradation in spectral resolution, pupil responses grew larger and at faster rates, suggesting an increase in listening effort while understanding speech. In the current study, we assessed listening effort during voice discrimination instead of speech intelligibility, thus our results extend on the findings of Winn et al. (2015), where we observed significantly larger pupil dilation responses with 12-band vocoder manipulations compared to non-vocoded speech while differentiating voices.

We expected acoustic/linguistic variability across items to yield larger pupil responses and accordingly, voice discrimination to be effortful, compared to when fixed items were presented. As mentioned above, research showed that introducing variability across items yields larger JNDs compared to when fixed items were presented during voice discrimination (Koelewijn et al. 2021a). Based on those findings, we expected that to perform optimally, voice discrimination with acoustic/linguistically variable items would require more effort, compared to when item variability is controlled. Contrary to our expectations, PPD was not significantly different when acoustic/linguistically variable or fixed items were presented on a voice discrimination task. Instead, item variability manipulations significantly affected the pupil response during the baseline period, where in comparison to fixed items, variable items yielded significantly larger pupil dilation responses. This suggests anticipated task difficulty before the onset of the stimuli presentation with variable items (Koelewijn et al. 2014, 2015). Pupil responses to item variability during baseline also depended on the vocoder manipulations, as was indicated by an interaction. Research suggests that activity in the Locus Coeruleus (LC), which, in relation to behavior, exhibits phasic and tonic modes of function, is linked to the changes in the pupil diameter (Aston-Jones & Cohen 2005; Koelewijn et al. 2014, 2015). According to the adaptive gain theory (Aston-Jones & Cohen 2005), while the phasic mode is related to task engagement and performance optimization, the tonic mode is more susceptible to distractibility and disengagement from the task. The transition between phasic and tonic modes is related to cost-benefit computations and a shift from phasic mode to tonic mode can result from a change in reward and diminishment of utility for a given task (Aston-Jones & Cohen 2005). Aston-Jones & Cohen (2005), measured baseline and task-evoked pupil responses in a tone discrimination task, where utility diminishes. In the task, rewards for correct responses increased with increasing task difficulty. However, the increase in task difficulty resulted in more errors after several trials, which decreased the reward. The authors found that early in each trial there were large phasic pupil dilations and pupil diameters during the baseline period were larger when the task became too difficult and utility declined. Additionally, baseline pupil diameters were largest when participants chose to abandon the task. Thus, the phasic mode is related to the moderate baseline firing rate of LC and larger responses to the task-related activities, whereas the tonic mode corresponds to high baseline firing of LC and larger baseline pupil diameters (Van der Meer et al. 2010). Based on this theory, our results suggest that voice cue discrimination with variable items brings listeners more into a tonic (exploratory) mode and away from a phasic (task engaged) mode compared to fixed items. Therefore, with acoustic/linguistic variability, especially when items were vocoded, the voice discrimination task was likely anticipated to be more difficult. This anticipation that occurred during the hardest condition in this experiment likely contributed to disengagement from the task, indicated by increased cognitive load before the stimulus onset and not during the task.

Our findings suggest that with a short-term implicit voice training, voice discrimination among vocoder-degraded speech became less effortful when listeners were trained with one of the voices. Considering that understanding vocoder-degraded speech, which reflects some aspects of cochlear implant listening, is effortful (Winn et al. 2015), and requires more attentional resources than listening to unprocessed speech (Wild et al. 2012), it is very important to find means to reduce this elevated listening effort. Cognitive resources are limited (Kahneman 1973), and reduced listening effort would leave more cognitive resources to be available for other tasks such as understanding speech and remembering what was being said (Rabbitt 1966). Having spare cognitive resources could especially be advantageous for hearing-impaired listeners and implant users, that could prevent fatigue and occupational difficulties associated with the increased listening effort in these populations (Hétu et al. 1988; Kramer et al. 1997, 2006; Hicks & Tharpe 2002). An increase in listening effort is also associated with slower processing speed (Houben et al. 2013) which is also detrimental for real-time speech processing that is necessary for everyday conversations (Başkent et al. 2016b). In our study, even when there was no clear benefit in behavioral measures of sensitivity, listening to a vocoded speech from trained voices altered the time course of pupil dilation, implying reduced cognitive load, while detecting small voice cue changes. To our knowledge, our study is the first that shows pupil response to be sensitive to perceptual differences at the early stage of voice cue processing, when the speech signal was vocoder degraded. This effect, observed in the absence of a behavioral training benefit, is exemplary of the importance of autonomic measures like pupillometry when it comes to investigating compensatory processes such as voice training that may not necessarily show an effect at the behavioral performance level.

### Implications for Future Research

While vocoder degradations only partially simulate degradations of electric stimulation, we can still speculate on the potential implications of our findings for implant users. Overall, implicit voice training, which is similar to real-life situations where voice familiarity is established by conversing with a specific person for an amount of time, has shown to be of benefit at the level of voice cue perception, by reducing listening effort, when speech is vocoder degraded. Therefore, even when short-termed, story narration from one voice can potentially benefit implant users by reducing listening effort and spare cognitive resources could further contribute to performance at the behavioral level through improvement in intelligibility, voice discrimination, and memory of what was being said, in real-life listening situations. Future research can focus on how to optimize voice training to improve voice discrimination performance with vocoder-degraded speech. Voice training can also incorporate vocoded speech.

Previously, changing *f*_*o*_ and *vtl* together and not individually was shown to produce largest improvements in speech intelligibility (Darwin et al. 2003). Additionally, manipulating *f*_*o*_ and *vtl* individually would have increased the testing time significantly, and in pupillometry experiments, testing time should be carefully considered as longer experiments might lead to fatigue, which can affect the results. Therefore, in our study, we chose to manipulate *f*_*o*_ and *vtl* voice cues together and not individually. However, it is important for future studies to investigate if individual manipulations of *f*_*o*_ and *vtl* lead to a voice training benefit to better understand the mechanisms of perception of individual voice cues.

Further, voice training methods can be tested if, in addition to voice discrimination, voice training would also improve speech intelligibility in speech-on-speech situations. Insights on the effects of voice training from studies with normal-hearing listeners using vocoder-degraded speech can be later tested with actual implant users. With implant users, results could be different, since implant users have to listen to degraded speech all the time, unlike the normal-hearing listeners of the present study, or listening effort benefits may be even more substantial with implant users. Longer sessions of auditory training involving audiobook listening may produce benefits to be seen also at the behavioral level (Jiam et al. 2019).

## CONCLUSIONS

In this study, we showed that voice cue discrimination was hindered by vocoding and acoustic/linguistic variability whereas voice training did not affect sensitivity to small changes in *f*_*o*_*+vtl* voice cues. Therefore, our knowledge regarding the role of voice training on the perceptual discriminability of *f*_*o*_*+vtl* voice cues is not yet comprehensive. Nevertheless, with vocoder degradation, voice cue discrimination was less effortful when speech was uttered by a trained voice than by an untrained voice. As previous research mainly focused on the intelligibility benefits of voice training, our findings provide a new insight on benefits on voice discrimination that can be obtained from listening to trained voices, that is, a reduced listening effort in the presence of vocoder degradation.

## ACKNOWLEDGMENTS

The authors would like to thank Etienne Gaudrain for his contributions, Terrin Tamati for insightful discussions regarding the study design and VICI project Scientific Advisory Board members Carolyn McGettigan, Matt Davis and Jan Wouters for earlier brainstorming conversations on the project; Anne Caclin, Richard McWalter, Mengchao Zhang, Daniel Oberfeld-Twistel and Anna Dietze for providing insightful reviews on an earlier version of this manuscript presented at the 19th International Symposium on Hearing (ISH 2022). We would also like to thank the Center for Information Technology of the University of Groningen for their support and for providing access to the Peregrine high performance computing cluster.
